# Prioritizing Training Needs of School Health Staff: The Example of Vietnam

**DOI:** 10.3390/ijerph17155563

**Published:** 2020-08-01

**Authors:** Sookyung Kim, Hyeonkyeong Lee, Hyeyeon Lee, Bui Thi Thanh Loan, Le Thi Thanh Huyen, Nguyen Thi Thanh Huong

**Affiliations:** 1 College of Nursing, Yonsei University, Seoul 03722, Korea; sookyungkimm@gmail.com (S.K.); leeleah86@gmail.com (H.L.); 2 College of Nursing and Mo-Im Kim Nursing Research Institute, Yonsei University, Seoul 03722, Korea; 3 Head of Department of Basic Sciences, Quang Tri Medical College, Quang Tri Province 520000, Vietnam; loanpepper79plus@gmail.com; 4 College Clinic, Quang Tri Medical College, Quang Tri Province 520000, Vietnam; huyendove@gmail.com; 5 College of Health Sciences, Vin University, Hanoi 100000, Vietnam; huong.ntt@vinuni.edu.vn

**Keywords:** global health, developing countries, health personnel, health promotion, needs assessment, school nursing, training

## Abstract

Competencies of school health staff (SHS) members, including school nurses, are crucial to improving child and adolescent health. In Vietnam, although SHS members are dispatched to schools, they have limited training opportunities. This study identified SHS members’ training needs in a province of Vietnam. A cross-sectional, online survey was conducted with 204 SHS members. The performance and importance of SHS members’ competencies were measured using 59-items and rated by a 5-point Likert scale. SHS members’ training priorities were analyzed using the Borich Needs Assessment and the Locus for Focus model. Controlling infectious disease was the highest training priority while implementing health promotion programs was of relatively low priority. The high-priority training needs identified could be rendered mandatory in policy for continuing education of SHS members. Awareness of the importance of health promotion, which has been emphasized globally, should also be promoted via school health policy. These findings could guide development of future training programs for SHS members.

## 1. Introduction

Establishing children and adolescents’ engagement in self-care is crucial to ensure their health in adulthood and ultimately reduce global health inequality [1]. Earlier studies have provided evidence that risky behaviors established during adolescence can continue into adulthood, thereby becoming several leading causes of mortality and morbidity [2]. Attention has been directed toward adolescence in low-and middle-income countries (LMICs) as part of their commitment to achieve the 2030 Sustainable Development Goals (SDGs) of ensuring healthy lives and promoting well-being for all, at all ages, and decreasing the incidence of non-communicable diseases, which represents the leading cause of preventable mortality [3]. As schools provide care and education for students for long periods, school is an important setting within which to promote adolescents’ health [1]. Well-designed school-based health interventions enable students to build competencies to prevent disease and promote health [4,5]. However, the school health staff (SHS) members responsible for school health service and education in LMICs have limited capacity. Therefore, global collaborative efforts are essential to improve key human resources on the frontline of ensuring students’ health; implementation of best practices across countries could represent an improved strategy.

SHS members play a key role in identifying unmet health needs of school-aged children and promoting health in schools. It is essential to strengthen the ability of SHS members to perform their roles adequately. SHS members in LMICs rarely have opportunities to engage in systematic education and training, and there is considerable variation in their competency [6]. In many countries, school nurses play an important role in promotion and prevention programs for school health, but in LMICs, the proportion of SHS members who are skilled health professionals is low [7]. In Vietnam, SHS members, including assistant doctors or nurses, are assigned to individual schools and usually attend a 1-day training session annually, but this does not occur regularly (Tien Le Thi Huong, personal communication, 26 July 2018). The role of SHS members in Vietnam is specified as “health records management of students and teachers, health education, first aid, care of general illness, and management of health equipment” [8]. However, systematic school health education and activities would likely be inadequate for students unless the practical competencies of SHS members are developed through continuing training. Thus, it is necessary to identify SHS members’ training needs prior to the development of training programs to improve their capacity. Accordingly, the purpose of this study was to identify SHS members’ training needs in a province of Vietnam through a global collaboration project.

## 2. Materials and Methods 

### 2.1. Study Design and Population

This study used a cross-sectional, descriptive design. It was conducted in Quang Tri province, one of 58 provinces in Vietnam with a population of approximately 650,000 and an area of 4746 km^2^. Quang Tri province is located in central north Vietnam and consists of two urban towns and eight rural districts. As the corresponding author’s university had previously collaborated with a college in Quang Tri province for several years, this study was conducted in the province to develop a future training program for SHS members. An SHS member qualified as an assistant doctor or nurse is assigned to each school. In some schools, non-health professionals are responsible for practical work as SHS members. The study targeted an entire sample of 243 SHS members of all schools, including non-health professionals, responsible for school health in primary and secondary schools in Quang Tri province in Vietnam. Out of 243 SHS members, 233 who had valid email addresses provided by Department of Education Training (DOET) in Quang Tri province were targeted in this study. The response rate was 96.1% (*N* = 224). Twenty SHS members were excluded because of missing data, outliers, or duplicate submissions. Ultimately, the data of 204 SHS members were included in the analysis.

### 2.2. Measures

**Figure 1:**
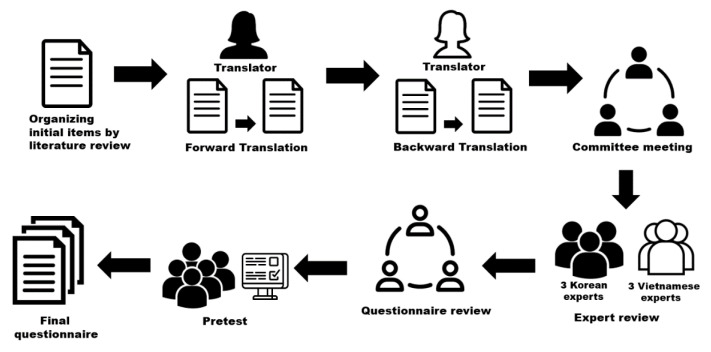
Questionnaire development and translation process.

The training needs assessment questionnaire (TNAQ) developed for the current study consists of three parts: perceived importance of competency for SHS members (59 items), perceived performance of competency for SHS members (59 items), and sociodemographic information (14 items). The competency items for SHS members were developed in multiple steps (Figure 1): organizing initial items pertaining to SHS members’ competencies according to a literature review, back translation, expert review using a content validity index, pretest, and finalization. The items were scored using a five-point Likert scale ranging from 1 (not important/confident) to 5 (very important/confident). The Cronbach’s alpha value assessing internal consistency reliability was 0.98 in this study.

#### 2.2.1. Organizing Initial Items

Initial items pertaining to SHS members’ competencies in Vietnam were developed using eight domains from the health teachers’ job analysis [9], which is consistent with roles of SHS member proposed by school health law in Korea and Vietnam. One domain of “establishing healthy and safe physical environment” based on the Monitoring and Evaluation Guidance for School Health Programs [10]. The initial questionnaire consisted of 59 items pertaining to SHS members’ training needs in nine domains: providing emergency care (Domain 1), providing health education (Domain 2), operating the school health room (Domain 3), implementing health screening for students (Domain 4), controlling infectious diseases (Domain 5), establishing a healthy and safe physical environment (Domain 6), providing health counseling (Domain 7), implementing health promotion programs (Domain 8), and developing professionalism (Domain 9).

#### 2.2.2. Back Translation

Back translation was used to develop culturally appropriate measurements [11]. A bilingual translator, who was fluent in English and Vietnamese and understood school health in Vietnam, translated the English version into Vietnamese. Another English-Vietnamese translator, who had not seen the original version, translated the Vietnamese version back into English. Research team members compared both versions in the original language for inconsistencies, mistranslations, and meaning. In the final step, a committee meeting was held between the research team members and another English-Vietnamese translator who was not involved in the previous steps. 

#### 2.2.3. Expert Review Using a Content Validity Index

Content validity was assessed by three school health experts in Korea and three school health experts in Vietnam, consistent with the criteria outlined by Lynn [12]. Items with a content validity index of less than 0.80 were reviewed by three authors (SK, HL, and HYL) to determine whether the item was necessary for the purpose of the study. Two items were deleted (“teaching cardiopulmonary resuscitation and first aid” and “providing group education,” as they overlapped with other items), and two items were added (“providing counseling to students with mental or psychological trauma,” as proposed by the DOET in Quang Tri province, and “planning health promotion programs,” as suggested by a Korean expert). One item was modified from “providing counseling to students with abnormal health problems” to “providing counseling to students with health problems.” This resulted in a total of 59 items for a pretest.

#### 2.2.4. Pretest and Finalization

A pretest was conducted with five SHS members who were working in schools in the research area, via an online survey [11]. No items were considered difficult to understand in a Vietnamese context. Ultimately, 59 items probing SHS members’ competencies were included in the final TNAQ.

### 2.3. Data Collection

In cooperation with Quang Tri Medical College and the DOET, an online survey was conducted. Survey announcements were sent to all SHS members via email. After reading an explanation of the study that was provided when accessing the online survey, SHS members who wished to participate clicked a button to provide consent. Twenty days later, the DOET sent a reminder email to consenting SHS members. Data were collected from July 5 to 28, 2019.

### 2.4. Statistical Analysis

Data were analyzed using IBM SPSS Statistics for Windows, Version 25.0 (IBM Corp., Armonk, NY, USA). Participants’ characteristics were analyzed using means, standard deviation (SDs), frequencies and percentages. A t-test was performed to compare SHS member’ performance levels according to general characteristics. Perceived performance and perceived importance ratings provided by SHS member were analyzed using means, SDs, and paired t-tests. SHS member’ training needs were identified using the Borich Needs Assessment [13] and the Locus for Focus model [14]. Borich Needs Assessment identified the “what is” (performance level) and “what should be” (importance level), and weighted the “what should be” (importance level) of each item to determine the priority of items [13]. The priority of training needs was represented by an x-y plane using the Locus for Focus model [14]. The median value of the x axis shows the average score of the importance level, while that of the y axis shows the average score of discrepancy between the importance and performance level (i.e., first quadrant is higher than the average importance level and higher than the average discrepancy between the two levels). The number of items having priority in the Borich Needs Assessment can be decided using the number of items included in the first quadrant (in the right upper quadrant) of the Locus for Focus model. Top ranking consistent items of the Borich Needs Assessment and items in the first quadrant from the Locus for Focus model represented the highest priority of training needs for SHS members [15].

### 2.5. Ethics

The study was approved by the institutional review board at the institution with which the first author was affiliated (IRB NO. Y-2019-0004). Before the online survey began, the study purpose, anonymity, and confidentiality were explained. Participants were advised that clicking the “Next” button indicated consent to participate.

## 3. Results

### 3.1. Participants’ Characteristics and Differences in Performance

Participants’ mean age was 34.28 years (SD = 6.76). Approximately 45.1% of participants were assistant doctors or nurses, and 54.9% reported other professions (e.g., accountant and librarian); 55.9% and 44.1% were primary and secondary school staff members, respectively. About 15.7% of the participants worked in schools in urban towns in Quang Tri province, and 28.9% of schools contained minority students. Regarding characteristics related to school health, 66.7% of health education providers were SHS members and 91.2% of schools provided regular heath education. Of the participants, 17.2% did not receive training regarding school health within the past two years (Table 1).

Perceived performance levels of nurses or physicians’ assistants (Mean = 3.78) were significantly higher than those of non-health professionals (Mean = 3.21; t = 5.06, *p* < 0.001). Perceived performance levels of participants working in secondary schools (Mean = 3.64) were significantly higher than those of participants working in primary schools (Mean = 3.34; t = −2.59, *p* = 0.01). Participants who received training in school health within the past two years (Mean = 3.55) reported significantly higher performance than those who had not received such training (Mean = 3.09; t = 2.92, *p* = 0.004). There was no significant difference in SHS members’ performance according to district (t = 0.33, *p* = 0.744) or whether schools contain minority students (t = −0.21, *p* = 0.837; Table 1).

### 3.2. SHS members’ Training Needs

Participants’ mean performance score over all items was 3.48 (SD = 0.86), whereas the overall average importance score was 4.35 (SD = 0.50). The average scores significantly differed between performance and importance (t = 13.65, *p* < 0.001) and all 59 items exhibited statistically significant differences between ratings of performance and importance (Table 2).

The average score for SHS members’ training needs was 3.79 according to the Borich Needs Assessment. Borich needs scores for all items in Domain 5 (controlling infectious disease) were higher than the average Borich needs score. Borich Needs scores for ≥ 50% of items were higher than the average score for Domains 1 (providing emergency care), 4 (implementing health screening for students), and 9 (developing professionalism; Table 2).

**Figure 2:**
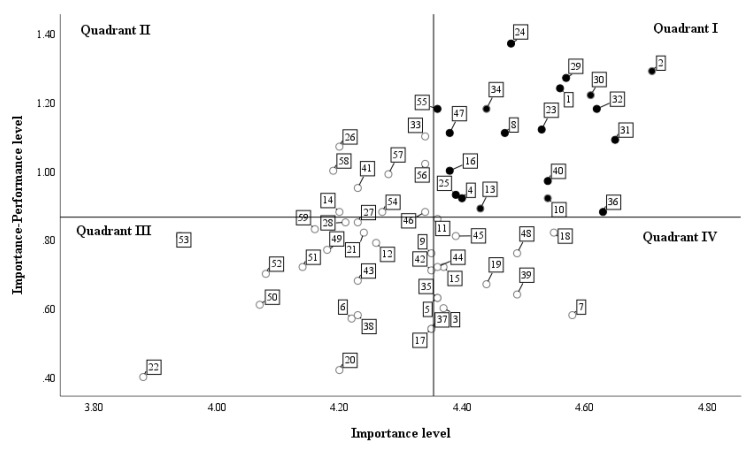
Priority of training needs in the locus for focus model.

There was considerable discrepancy in importance and performance in the locus for focus model (Figure 2). The first quadrant represented the highest priority, as the importance and discrepancy between importance and performance were higher than average. In total, 19 priority training needs were included in the first quadrant and eight of nine domains (all but Domain 8, implementing health promotion programs). Domain 5 (controlling infectious diseases), Domain 1 (providing emergency care), and Domain 4 (implementing health screening for students) included numerous items pertaining to priority training needs (Table 3). Fourteen items were both in the 19 top priority items in the Borich Needs Assessment and in the first quadrant of the Locus for Focus model. Ten items, which were derived from only one of the Borich Needs Assessment or the Locus for Focus model, were not given high priority in training needs (Table 3).

### 3.3. Consensus Regarding SHS Members’ Training Needs

An online consensus development panel was assembled to obtain agreement regarding training needs and share the survey results. In total, 93 SHS members who attended the 2019 annual SHS members’ training responded. All of the top 10 training needs for SHS members were agreed upon through consensus, with percentages ranging from 92.5% to 98.9%.

## 4. Discussion

This was a nurse-led global health project that aimed to identify the priority of training needs to strengthen the capacity of SHS members, who rarely have opportunities to continue professional development. It is worth mentioning that the cooperation of researchers from both countries was beneficial in conducting this needs assessment prior to designing a Vietnam-specific training program, as integrating the needs and circumstances of SHS members in Vietnam and would benefit from evidence accumulated pertaining to Korean school health teachers. As part of a global commitment to achieve SDG 3, the findings provide data to help establish training programs for SHS members, who play key roles in providing quality school health service and improving health knowledge and healthy behaviors of students in LMICs. The TNAQ will be useful in future research in LMICs to provide valid and reliable assessments of the performance and importance of SHS members’ activities.

The domain of “controlling infectious diseases” was identified as a top priority area for SHS members’ training, as lower performance than average was reported while the domain was considered of higher than average importance for all competency items but one. Specifically, “building a system of infectious disease control” and “monitoring and managing students with infectious diseases” represented the highest priority training needs. As suggested by Kim and colleagues [16], it is important to establish systems and action plans in schools that address outbreaks of pandemic diseases. In Korea, school health teachers plan and take action in response to infectious diseases in schools appropriately according to the situation, by following an infectious disease manual [17] distributed by the Ministry of Education. For example, in general cases of infectious disease, school health teachers confirm vaccination completion of students and encourage vaccination for unvaccinated students; provide preventive education regarding infectious disease for students, parents, and school personnel; and monitor students at-risk of infectious disease and report infected students to public health centers [17]. During infectious disease outbreaks such as the Middle East Respiratory Syndrome (MERS), school health teachers are required to coordinate school health services, develop plans for distributing infection-control supplies, construct referral systems to public health centers and local clinics for screening, and provide health education for both parents and students for preventing infectious disease [18]. In resource-limited communities in LMICs, SHS members are expected to play a vital role in responding to both infectious diseases in general and outbreaks, which highlights the need for training programs.

Providing emergency care (Domain 1) included the three top-ranking items requiring priority training. According to recommendations on the role of school nurses during emergencies by the National Association of School Nurses (NASN) [19], training content for school nurses should include identifying hazards, serving on planning groups, building emergency response plans, and coordinating first aid response teams; these were identified in the current study as items that should be prioritized in SHS members’ training. Where resources and accessibility of medical facilities are limited in LMICs, it would be necessary to strengthen the capacity of SHS members to appropriately manage medical emergencies in schools. NASN emphasized SHS members as key persons to act as liaisons between community resources [19]. Thus, SHS members should be trained to organize community networks and link community transportation resources for urgent patient transfers. In a previous study [20], school nurses who were well-trained in medical emergency response plans were confident when managing head/neck injury of students and determining the availability of emergency equipment. Note that more than half of the SHS members in the current study were non-professionals who reported low competency in several skills. Therefore, considerable education regarding how to address emergencies should be provided to SHS members.

In addition to infectious and emergency care, the SHS participants in this study exhibited high training needs in the areas of health screening, counseling students with mental or psychological trauma, and protecting children from danger from road traffic, animals, and fire. According to the American Academy of Pediatrics on School Health [21], schools should provide regular and developmental health screening for vision, oral health, hearing, height, and weight for secondary prevention in schools. It is interesting to consider counseling students with mental or psychological trauma and protecting children from danger from road traffic, animals, and fire. In a recent study of Vietnamese adults, 46.9% had been exposed to a traumatic event in their life [22]. Children of parents who have experienced traumatic events are likely to experience psychological problems, as evidenced by findings that children of war veterans with post-traumatic stress disorder (PTSD) experience more psychological issues than do children of veteran fathers without PTSD [23]. In addition, motorcycles are a major form of transportation in Vietnam, and mortality of children and adolescents due to motorcycle accidents is high [24]. Further, road traffic injuries are common among older adolescents and those who consume alcohol before riding motorcycles [24]. This suggests that training for SHS members should include strategies to educate adolescents regarding the risks associated with motorcycling.

No items were identified as being of high priority in “implementing health promotion programs” (Domain 8) in the current study. The results are consistent with low levels of awareness of the importance of health promotion [25] and promoting healthy behaviors among adolescents [26] in LMICs. However, the WHO has emphasized that schools are of strategic value for guiding preventive health behaviors as a key to health promotion [27]. The WHO has further stated that children and adolescents are the most important population for fostering the adoption of healthy lifestyles in the future [28]. In addition, as school-based health promotion programs exert positive effects on children’s and adolescents’ health [29,30], consideration should also be given to efforts to increase awareness about these issues among SHS members in the process of developing the training program.

Providing continuous training for health professionals in areas with shortages of, and low accessibility to, medical resources is crucial [31] and would ultimately exert a significant effect on children’s and adolescents’ health [21]. The current study showed that non-health professionals (e.g., accountants and librarians) in charge of school health demonstrated significantly poorer performance than did health professionals, indicating an urgent need to develop the capacity of the former to provide school health. It is noteworthy that increased training and opportunities for continuous professional development could reduce variation in competency among types of SHS professional [7].

The study was subject to some limitations. It is difficult to generalize the results regarding training needs to all SHS members in LMICs because the study was conducted in a single province of Vietnam. In addition, training needs were examined only via an online survey. Further research involving qualitative needs assessment is required to explore the training context in-depth.

The current findings support the need for a policy of mandatory training for school health professionals, including nurses. Training needs for health promotion were of low priority, but there is a need for political support for long-term health promotion programs for young people and efforts to increase awareness regarding the importance of this issue. This study was conducted to identify training needs of all SHS members of a province in Vietnam, one of the LMICs, in close cooperation with a local college and provincial education department. Therefore, this study’s strength is that the results can be practically applied to training programs for SHS members in the future. It is important to assess SHS members’ performance in each country at a local level and provide them with needs-based appropriate training. The current findings could be of utility for other developing countries in research and policy pertaining to SHS members.

## 5. Conclusions

This study consisted of a Korean-Vietnamese collaborative project to identify high-priority training needs of SHS members in a province in Vietnam. The findings provide empirical evidence that could inform the development of a Vietnam-specific training program for SHS members. SHS members’ competencies in LMICs with limited resources could exert a significant effect on young people’s health. Training content should be organized to control infectious diseases and enhance the ability of SHS members to manage emergency care in school settings. In addition, long-term health promotion should receive focus.

## Figures and Tables

**Table 1:** Participants’ Characteristics and Differences in School Health Staff Members’ Performance (*N* = 204).

Characteristics	Categories	Mean ± *SD/n* (*%*)	Performance Level	
			Mean ± *SD*	*T (p)*
Age	34.28 ± 6.76			
Gender	Male	28 (13.7)	3.73 ± 0.81	1.69 (0.092)
	Female	176 (86.3)	3.43 ± 0.87	
Type of school health staff	Assistant doctor, Nurse	92 (45.1)	3.78 ± 0.64	5.06 (<0.001)
	Other	112 (54.9)	3.21 ± 0.95	
Type of school	Primary	114 (55.9)	3.34 ± 0.93	−2.59 (0.010)
	Secondary	90 (44.1)	3.64 ± 0.76	
Place of residence	Urban	32 (15.7)	3.51 ± 0.87	0.33 (0.744)
	Rural	172 (84.3)	3.46 ± 0.87	
Schools with minority students	Yes	59 (28.9)	3.45 ± 0.89	−0.21 (0.837)
	No	145 (71.1)	3.47 ± 0.86	
Type of health education provider	School health staff	136 (66.7)	3.69 ± 0.78	5.51 (<0.001)
	Other	68 (33.3)	3.03 ± 0.88	
Regular health education at school	Yes	186 (91.2)	3.55 ± 0.83	4.08 (<0.001)
	No	18 (8.8)	2.70 ± 0.90	
School health training within the past 2 years	Yes	169 (82.8)	3.55 ± 0.83	2.92 (0.004)
	No	35 (17.2)	3.09 ± 0.98	

**Table 2:** School Health Staff Members’ Training Needs by Borich Needs Assessment (*N* = 204).

Item	Importance Mean ± *SD*	Performance Mean ± *SD*	t	Borich Needs Score	Rank
**Domain 1. Providing emergency care**					
1. Carrying out health assessment	4.56 ± 0.67	3.32 ± 1.20	14.63 ***	5.68	4
2. Administering first aid	4.71 ± 0.49	3.42 ± 1.12	16.32 ***	6.10	2
3. Providing mental support	4.37 ± 0.69	3.77 ± 0.94	8.26 ***	2.61	52
4. Administering first-aid medicine	4.40 ± 0.74	3.48 ± 1.18	10.41 ***	4.08	23
5. Guiding self-healthcare	4.36 ± 0.71	3.73 ± 0.96	8.21 ***	2.78	49
6. Guiding follow-up management	4.22 ± 0.77	3.65 ± 0.96	7.23 ***	2.40	55
7. Making requests to medical institutions	4.58 ± 0.61	4.00 ± 0.89	8.05 ***	2.63	51
8. Building an emergency medical system	4.47 ± 0.65	3.36 ± 1.08	14.15 ***	4.95	11
**Domain 2. Providing health education**					
9. Establishing annual operation plans for health education curriculum	4.35 ± 0.68	3.59 ± 1.00	10.22 ***	3.28	38
10. Teaching students about health	4.54 ± 0.61	3.62 ± 0.94	12.79 ***	4.18	20
11. Teaching teachers about health	4.36 ± 0.69	3.50 ± 1.00	10.71 ***	3.78	28
12. Teaching parents about health	4.26 ± 0.83	3.47 ± 1.02	8.70 ***	3.40	37
13. Offering health information	4.43 ± 0.65	3.54 ± 0.97	11.72 ***	3.95	25
14. Providing broadcasting education	4.20 ± 0.78	3.32 ± 1.12	10.34 ***	3.66	30
**Domain 3. Operating the school health room**					
15.Establishing annual operation plans for health management	4.37 ± 0.66	3.65 ± 1.01	10.22 ***	3.17	40
16. Producing budgets	4.38 ± 0.64	3.38 ± 1.06	12.38 ***	4.38	17
17. Managing a daily record of health work	4.35 ± 0.65	3.81 ± 0.96	7.67 ***	2.35	56
18. Managing medicines	4.55 ± 0.62	3.73 ± 1.06	10.60 ***	3.75	29
19. Managing medical instruments	4.44 ± 0.66	3.77 ± 1.05	8.69 ***	2.96	45
20. Taking care of official documents	4.20 ± 0.76	3.78 ± 0.93	5.68 ***	1.73	58
21. Obtaining necessary or suggested items	4.24 ± 0.74	3.42 ± 1.05	9.84 ***	3.47	34
22. Managing other related documents	3.88 ± 0.85	3.48 ± 0.96	5.08 ***	1.54	59
**Domain 4. Implementing health screening for students**					
23. Investigating health conditions	4.53 ± 0.66	3.41 ± 1.11	13.14 ***	5.09	9
24. Implementing health screening	4.48 ± 0.67	3.11 ± 1.19	14.76 ***	6.13	1
25. Managing children requiring protection	4.39 ± 0.68	3.46 ± 1.03	11.64 ***	4.09	21
26. Providing aftercare based on results	4.20 ± 0.86	3.13 ± 1.14	12.06 ***	4.47	14
27. Managing record of results	4.23 ± 0.85	3.38 ± 1.07	10.13 ***	3.61	31
28. Using statistical results	4.21 ± 0.86	3.36 ± 1.08	9.52 ***	3.57	32
**Domain 5. Controlling infectious diseases**					
29. Building a system of infectious disease control	4.57 ± 0.62	3.30 ± 1.16	14.76 ***	5.80	3
30. Monitoring infectious diseases	4.61 ± 0.58	3.39 ± 1.19	14.40 ***	5.65	5
31. Providing preventive education on infectious disease	4.65 ± 0.54	3.56 ± 1.10	13.77 ***	5.06	10
32. Managing infected students	4.62 ± 0.59	3.44 ± 1.13	14.30 ***	5.46	6
33. Checking for necessary vaccinations	4.34 ± 0.79	3.24 ± 1.21	12.26 ***	4.77	13
34. Monitoring epidemiologically infectious environments	4.44 ± 0.70	3.26 ± 1.13	13.83 ***	5.22	7
**Domain 6. Establishing a healthy and safe physical environment**					
35. Assessing and managing available latrines for boys and girls	4.36 ± 0.71	3.73 ± 0.91	9.09 ***	2.78	50
36. Assessing and managing safe drinking water	4.63 ± 0.58	3.75 ± 0.98	12.01 ***	4.09	22
37. Assessing and managing available hand washing facilities	4.35 ± 0.68	3.81 ± 0.91	7.42 ***	2.35	57
38. Assessing and managing well-constructed and maintained learning areas and spaces	4.23 ± 0.75	3.65 ± 0.91	8.40 ***	2.47	54
39. Assessing and managing garbage removal from school grounds	4.49 ± 0.66	3.85 ± 0.86	9.36 ***	2.88	46
40. Protecting children from danger from road traffic, animals, and fire	4.54 ± 0.75	3.57 ± 1.01	12.93 ***	4.38	16
41. Assessing and managing endocrine disrupting chemicals (e.g., plastics, pesticides, detergent, Styrofoam, batteries)	4.23 ± 0.87	3.28 ± 1.13	11.03 ***	4.02	24
**Domain 7. Providing health counseling**					
42. Teaching putting healthy lifestyle habits into practice	4.35 ± 0.74	3.64 ± 0.95	9.11 ***	3.07	43
43. Providing health information to satisfy counseling needs	4.23 ± 0.74	3.55 ± 0.95	9.25 ***	2.88	47
44. Counseling students about health	4.36 ± 0.69	3.64 ± 0.97	9.64 ***	3.14	42
45. Providing counseling to students with health problems	4.39 ± 0.71	3.58 ± 0.98	10.67 ***	3.55	33
46. Guiding student with their family on health problems	4.34 ± 0.72	3.46 ± 1.05	10.82 ***	3.85	26
47. Providing counseling to students with mental or psychological trauma	4.38 ± 0.74	3.27 ± 1.14	12.61 ***	4.85	12
48. Making requests to related institution	4.49 ± 0.66	3.73 ± 0.93	11.12 ***	3.41	36
**Domain 8. Implementing health promotion programs**					
49. Understanding needs	4.18 ± 0.74	3.41 ± 1.03	10.27 ***	3.22	39
50. Setting order of priority	4.07 ± 0.81	3.46 ± 0.98	8.39 ***	2.49	53
51. Planning health promotion programs	4.14 ± 0.75	3.42 ± 1.04	10.53 ***	2.98	44
52. Utilizing human and material resources	4.08 ± 0.78	3.38 ± 0.98	9.15 ***	2.88	48
53. Running health promotion programs	3.94 ± 0.83	3.13 ± 1.12	9.60 ***	3.17	41
54. Evaluating results	4.27 ± 0.71	3.39 ± 1.00	11.50 ***	3.79	27
**Domain 9. Developing professionalism**					
55. Receiving teacher training	4.36 ± 0.74	3.18 ± 1.16	13.73 ***	5.15	8
56. Searching for the latest medical information	4.34 ± 0.72	3.32 ± 1.11	12.67 ***	4.43	15
57. Searching for the latest teaching materials	4.28 ± 0.74	3.29 ± 1.12	12.26 ***	4.24	18
58. Developing health education materials	4.19 ± 0.82	3.19 ± 1.09	11.67 ***	4.19	19
59. Participating in job-related organization	4.16 ± 0.79	3.33 ± 1.09	10.51 ***	3.47	35
Total	4.35 ± 0.50	3.48 ± 0.86	13.65 ***	3.79	

*** *p* < 0.001.

**Table 3:** High Priority Training Needs for School Health Staff Members According to Borich Needs Assessment and Locus for Focus Model.

Domain	Item	Rank(Borich Needs Assessment)	Quadrant(Locus for Focus Model)	High Priority
**4**	**24. Implementing health screening**	**1**	**Ⅰ**	**O**
**1**	**2. Administering first aid**	**2**	**Ⅰ**	**O**
**5**	**29. Building a system of infectious disease control**	**3**	**Ⅰ**	**O**
**1**	**1. Carrying out health assessment**	**4**	**Ⅰ**	**O**
**5**	**30. Monitoring infectious diseases**	**5**	**Ⅰ**	**O**
**5**	**32. Managing infected students**	**6**	**Ⅰ**	**O**
**5**	**34. Monitoring epidemiologically infectious environments**	**7**	**Ⅰ**	**O**
**9**	**55. Receiving teacher training**	**8**	**Ⅰ**	**O**
**4**	**23. Investigating health conditions**	**9**	**Ⅰ**	**O**
**5**	**31. Providing preventive education on infectious disease**	**10**	**Ⅰ**	**O**
**1**	**8. Building an emergency medical system**	**11**	**Ⅰ**	**O**
**7**	**47. Providing counseling to students with mental or psychological trauma**	**12**	**Ⅰ**	**O**
5	33. Checking for necessary vaccinations	13	Ⅱ	
4	26. Providing aftercare based on results	14	Ⅱ	
9	56. Searching for the latest medical information	15	II	
**6**	**40. Protecting children from danger from road traffic, animals, and fire**	**16**	**Ⅰ**	**O**
**3**	**16. Producing budgets**	**17**	**Ⅰ**	**O**
9	57. Searching for the latest teaching materials	18	II	
9	58. Developing health education materials	19	II	
2	10. Teaching students about health	20	Ⅰ	
4	25. Managing children requiring protection	21	Ⅰ	
6	36. Assessing and managing safe drinking water	22	Ⅰ	
1	4. Administering first-aid medicine	23	Ⅰ	
2	13. Offering health information	25	Ⅰ	

Note. Domain 1: Providing emergency care; Domain 2: Providing health education; Domain 3: Operating the school health room; Domain 4: Implementing health screening for students; Domain 5: Controlling infectious diseases; Domain 6: Establishing a healthy and safe physical environment; Domain 7: Providing health counseling; Domain 8: Implementing health promotion programs; Domain 9: Developing professionalism. Bold indicates the fourteen top priority items for training needs of school health staff.
